# Abnormal interhemispheric resting state functional connectivity in Parkinson’s disease patients with impulse control disorders

**DOI:** 10.1038/s41531-021-00205-7

**Published:** 2021-07-16

**Authors:** Caiting Gan, Lina Wang, Min Ji, Kewei Ma, Huimin Sun, Kezhong Zhang, Yongsheng Yuan

**Affiliations:** Department of Neurology, The First Affiliated Hospital of Nanjing Medical University, Nanjing, China

**Keywords:** Parkinson's disease, Parkinson's disease

## Abstract

Impulse control disorders (ICD) in Parkinson’s disease (PD) might be attributed to misestimate of rewards or the failure to curb inappropriate choices. The mechanisms underlying ICD were reported to involve the lateralization of monoamine network. Our objective was to probe the significant role of lateralization in the pathogenesis of ICD. Twenty-one PD patients with ICD (PD-ICD), thirty-three without ICD (PD-no ICD), and thirty-seven healthy controls (HCs) were recruited and performed T1-weighted, diffusion tensor imaging (DTI) scans and resting state functional magnetic resonance imaging (rs-fMRI). By applying the Voxel-mirrored Homotopic Connectivity (VMHC) and Freesurfer, we evaluated participants’ synchronicity of functional connectivity and structural changes between hemispheres. Also, tract-based spatial statistics (TBSS) was applied to compare fiber tracts differences. Relative to PD-no ICD group, PD-ICD group demonstrated reduced VMHC values in middle frontal gyrus (MFG). Compared to HCs, PD-ICD group mainly showed decreased VMHC values in MFG, middle and superior orbital frontal gyrus (OFG), inferior frontal gyrus (IFG) and caudate, which were related to reward processing and inhibitory control. The severity of impulsivity was negatively correlated with the mean VMHC values of MFG in PD-ICD group. Receiver operating characteristic (ROC) curves analyses uncovered that the mean VMHC values of MFG might be a potential marker identifying PD-ICD patients. However, we found no corresponding asymmetrical alteration in cortical thickness and no significant differences in fractional anisotropy (FA) and mean diffusivity (MD). Our results provided further evidence for asymmetry of functional connectivity in mesolimbic reward and response inhibition network in ICD.

## Introduction

Impulse control disorders (ICD), including pathological gambling (PG), binge eating (BE), compulsive shopping (CS), and hypersexuality (HS)^1^, are reported in 6.1–31.2% of Parkinson’s disease (PD) patients^2^, occurring primarily in patients treated with dopamine receptor agonists. PD patients with ICD are more prone to anxiety, depression, apathy and other mental disorders than patients without, and the severity of mental symptoms increases synchronously with the number of ICD^3^. ICD can lead to severe personal, family, economic and psychosocial burden, attracting more and more attention from researchers and clinicians.

Although the pathogenesis of ICD in PD patients remains unclear, emerging evidence supports that a complex multifactorial mechanism of serotoninergic and noradrenergic interaction was involved in the development of ICD, beyond the classical dopaminergic corticostriatal networks^4^. Namely, the presence of ICD could be ascribed to the engagement of reward and response inhibition networks, mediated by frontostriatal connectivity^5,6^. Susceptibility to the disease depends on related risk factors, such as younger age at onset, early PD onset, unmarried, cigarette smoking, caffeine use, treatment related, personal or family history, and the occurrence of motor complications^7^. Recently, a cross-sectional study suggested that right onset of PD might also be a risk factor for ICD^8^. Simultaneously, a DATSCAN imaging research supported that ICD symptoms were linked to the disruption of asymmetric molecular frontostriatal network centered on left basal ganglia, fitting well with the effect of left-hemisphere activity during reward processing^9^. These above findings implied that the laterality of dopamine and serotonin network might have significant influence on ICD. Based on these previous results, we intended to probe the important role of lateralization in the pathogenesis of ICD by more intuitively exploration of functional connectivity between hemispheres.

Thus, we adopted a validated analysis approach named Voxel-mirrored Homotopic Connectivity (VMHC), which could detect the altered interhemispheric connectivity via quantifying functional connections between each voxel in one hemisphere and its corresponding voxel in the other^10^. This is a well-established approach that has been widely applied in the studies of many diseases. Prior to our study, numerous neuropsychiatric diseases have been investigated using this method to reveal the functional coordination between hemispheres^11–13^. In general, a decrease in VMHC values represents a decrease in synchronicity between the two cerebral hemispheres, which could be interpreted as the possible presence of functional laterality. Furthermore, it is generally believed that changes of functional connectivity might be correlated with alterations of structure. Hence, we further analyzed whether the desynchronized brain regions of functional connections also showed asymmetry in cortical thickness. Meanwhile, diffusion tensor imaging (DTI) was also applied in our study to detect fiber tracts impairment.

## Results

### Demographic and clinical characteristics

Subjects were divided into three groups: 21 PD patients with ICD (PD-ICD), 33 PD patients without ICD symptoms (PD-no ICD), and 37 healthy controls (HCs). Clinical characteristics of PD subgroups and HCs are demonstrated in Table 1. No remarkable differences were observed in age, gender, and education. In relative to PD-no ICD group, the PD-ICD group revealed no significant differences in disease duration, age at onset, affected side at onset, Hoehn & Yahr stage (H-Y stage) scale, the Unified Parkinson’s Disease Rating Scale Section III (UPDRS-III), Barratt Impulsiveness Scale (BIS), total levodopa equivalent daily dose and levodopa equivalent daily dose of dopamine agonist. Unsurprisingly, ICD symptom severity worsened in PD-ICD group than PD-no ICD (p < 0.0001). Notably, 19 patients in PD-ICD group showed a single ICD and 2 cases had multiple impulse control and related disorders (ICD-RD). CS was the most frequent disorder (7/21, 33.3%), followed by BE (5/21, 23.8%), PG (5/21, 23.8%), HS (2/21, 9.5%), BE + CS + Hobbyism-punding (1/21, 4.8%), and CS + DDS (1/21, 4.8%). Mini Mental State Examination (MMSE) scores of HCs were significantly higher than that of PD-ICD and PD-no ICD groups (*p* = 0.015 and *p* = 0.006, respectively) after Bonferroni post-hoc test. Similarly, Hamilton Anxiety Scale (HAMA) and Hamilton Depression Scale-24 (HAMD-24) scores in HCs group were significantly lower in relative to PD-ICD and PD-no ICD groups (*p* < 0.0001).

**Table 1:** Demographic and clinical characteristics of the studied groups.

Variable	PD-ICD (*n* = 21)	PD-noICD (*n* = 33)	HCs (*n* = 37)	*p*	Post hoc (Bonferroni)
Age (y)	59.0 ± 9.6	61.7 ± 9.3	62.0 ± 5.6	0.378^b^	
Gender (M/F)	12/9	20/13	25/12	0.700^a^	
Education (y)	11.4 ± 3.1	10.3 ± 3.3	12.2 ± 3.8	0.085^c^	
Disease duration (y)	9.0 ± 5.2	7.1 ± 3.0	—	0.241^d^	
Age at onset (y)	50.9 ± 10.1	54.2 ± 9.7	—	0.235^e^	
Affected side at onset right% (n)	47.6% (10)	42.4% (14)	—	0.708^a^	
UPDRS part 3	20.3 ± 14.2	18.5 ± 11.3	—	0.518^d^	
H-Y stage	2.3 ± 0.7	2.2 ± 0.6	—	0.580^d^	
LEDD_TOTAL_ (mg)	770.7 ± 310.8	636.1 ± 271.5	—	0.085^d^	
LEDD_DA_ (mg)	90.5 ± 57.4	68.6 ± 50.7	—	0.150^e^	
DA %	85.70%	78.50%	—	0.523^a^	
QUIP-RS total score	13.0 ± 5.9	0	—	<0.0001^d, ***^	
BIS	33.1 ± 15.6	29.7 ± 15.3	—	0.495^d^	
MMSE	28.3 ± 1.1	28.0 ± 1.5	29.1 ± 0.9	0.002^c, **^	PD-ICD < HC (*p* = 0.015) PD-noICD < HC (*p* = 0.006)
HAMA	14.0 ± 8.9	11.2 ± 6.1	2.3 ± 3.3	<0.0001^c, ***^	PD-ICD < HC (*p* < 0.0001) PD-noICD < HC (*p* < 0.0001)
HAMD-24	12.6 ± 8.2	9.5 ± 7.0	1.7 ± 2.5	<0.0001^c, ***^	PD-ICD < HC (*p* < 0.0001) PD-noICD < HC (*p* < 0.0001)
FAB	16.2 ± 2.0	16.4 ± 2.5	17.0 ± 1.3	0.232^c^	

Values are presents as the mean ± standard deviation.

*PD* Parkinson’s Disease, *ICD* impulse control disorders, *HCs* healthy controls, *M* male, *F* female, *y* year, *UPDRS* Unified Parkinson’s disease rating scale, *H-Y stage* Hoehn and yahr clinical rating scale, *LEDD*_*TOTAL*_ total levodopa equivalent daily dose, *LEDD*_*DA*_ levodopa equivalent daily dose of dopamine agonist, *DA* dopamine agonist, *QUIP-RS* Questionnaire for Impulsive Compulsive Disorders in Parkinson’s Disease-Rating Scale, *BIS* Barratt Impulsiveness Scale, *MMSE* Mini Mental State Examination, *HAMA* Hamilton Anxiety Scale, *HAMD-24* Hamilton Depression Scale-24, *FAB* Frontal Assessment Battery.

^a^Chi-square test.

^b^One-way ANOVA.

^c^Kruskal-Wallis.

^d^Mann-Whitney U.

^e^Two-sample t-test.

*p < 0.05, **p < 0.01, ***p < 0.001.

### Voxel-mirrored homotopic connectivity

**Fig. 1:**
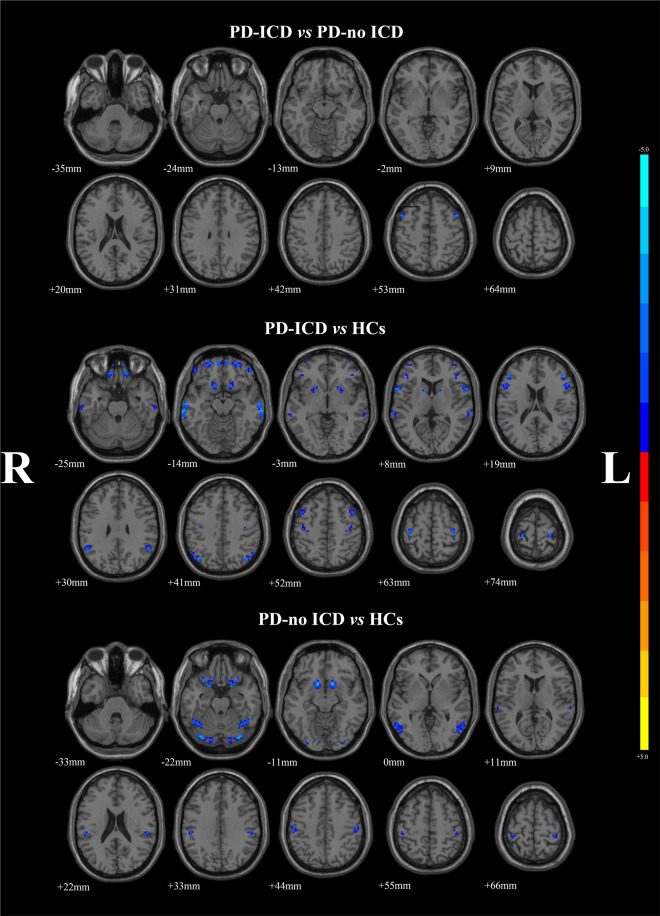
Statistical maps showing VMHC differences in different brain regions among three groups. The threshold for display was set to *p* < 0.01. *PD* Parkinson’s Disease, *VMHC* voxel-mirrored homotopic connectivity, *ICD* Impulse control disorders, *HCs* health controls, *R* right, *L* left.

Analysis of covariance (ANCOVA) was utilized to detected the VMHC differences among the three groups, following by the two-sample post hoc t-test. PD-ICD patients demonstrated reduced VMHC values in middle frontal gyrus (MFG) (MNI *x* = ± 45, *y* = 9, *z* = 57, T value = −3.6619) in relative to PD-no ICD group, after adjusting gender, age, education level, mean framewise displacement (FD), HAMA, HAMD-24 and MMSE scores. Compared with HCs, significant reduced VMHC values were founded in MFG (MNI *x* = ± 45, *y* = 9, *z* = 57, T value = −3.5509), inferior frontal gyrus (IFG) (MNI *x* = ± 60, *y* = 12, *z* = 9, T value = −3.6203), middle orbital frontal gyrus (MNI *x* = ± 24, *y* = 54, *z* = −18, T value = −3.8517) and superior orbital frontal gyrus (OFG) (MNI *x* = ± 15, *y* = 18, *z* = −18, T value = −3.4832), middle temporal gyrus (MNI *x* = ± 69, *y* = −30, *z* = −12, T value = −4.0977) and superior temporal gyrus (MNI *x* = ± 66, *y* = −27, *z* = 12, T value = −3.5061), caudate (MNI *x* = ± 12, *y* = 9, *z* = 6, T value = −3.3566), precentral (MNI *x* = ± 33, *y* = −21, *z* = 69, T value = −4.3051), and angular (MNI *x* = ± 42, *y* = −69, *z* = 42, T value = −3.4986) in the PD-ICD group. And PD-no ICD patients presented significant decreased VMHC values in the middle temporal gyrus (MNI *x* = ± 57, *y* = −63, *z* = 3, T value = −3.4022), superior orbital frontal gyrus (MNI *x* = ± 12, *y* = 24, *z* = −24, T value = −4.6984), superior cerebellum (MNI *x* = ± 33, *y* = −78, *z* = −21, T value = −3.9719), and postcentral gyrus (MNI *x* = ± 54, *y* = −21, *z* = 45, T value = −3.9756) compared to HCs (Fig. 1 and Table 2).

**Table 2:** VMHC differences among PD-ICD patients, PD-no ICD patients, and health controls during ON phase.

Brain regions (AAL)	Number of voxels	MNI Coordinates			BA	T Value
		X	Y	Z		
PD-ICD vs PD-no ICD						
Middle frontal gyrus	18	±45	9	57	6	−3.6619
PD-ICD vs HCs						
Middle frontal gyrus	30	±45	9	57	6	−3.5509
Middle orbital frontal gyrus	157	±24	54	−18	11	−3.8517
Superior orbital frontal gyrus	42	±15	18	−18	11	−3.4832
Caudate	25	±12	9	6	—	−3.3566
Inferior frontal gyrus (pars opercularis)	66	±60	12	9	6	−3.6203
Middle temporal gyrus	98	±69	−30	−12	21	−4.0977
Superior temporal gyrus	26	±66	−27	12	22	−3.5061
Precental gyrus	90	±33	−21	69	6	−4.3051
Angular gyrus	36	±42	−69	42	7	−3.4986
PD-no ICD vs HCs						
Superior orbital frontal gyrus	92	±12	24	−24	11	−4.6984
Superior cerebelum	175	±33	−78	−21	19	−3.9719
Middle temporal gyrus	76	±57	−63	3	37	−3.4022
Postcentral gyrus	172	±54	−21	45	3	−3.9756

The resulting statistical maps were set at *p* < 0.01 and corrected by AlphaSim. *VMHC* Voxel-mirrored Homotopic Connectivity, *PD* Parkinson’s disease, *ICD* Impulse control disorders, *HCs* healthy controls, *AAL* Anatomical Automatic Labeling, *BA* Brodmann area.

### Cortical thickness

The asymmetry index (AI) was not different between groups restricted to the brain regions showing VMHC differences after Bonferroni correction (Supplementary Table 1).

### Tract-based spatial statistics

Tract-based spatial statistics (TBSS) was applied to compare fiber tracts impairment across the whole brain among these three groups. However, no significant differences were detected among the groups (Supplementary Fig. 1).

### Correlation analysis

**Fig. 2:**
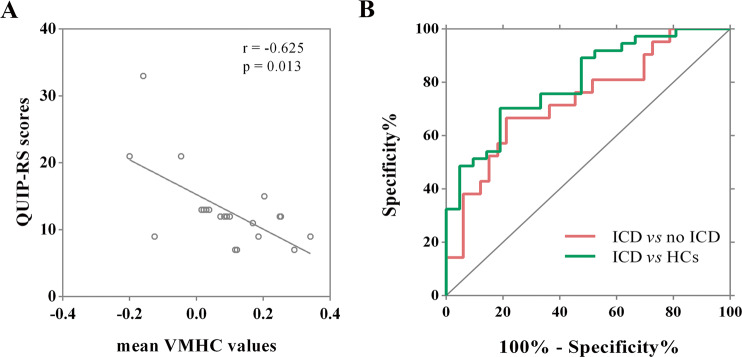
Correlation between VMHC values and QUIP-RS scores of PD-ICD patients and ROC analyses for differentiating different groups. Scatterplots demonstrated that there was a significant negative correlation between the mean VMHC values in the middle frontal gyrus and QUIP-RS scores in PD patients with ICD. The graph showed the results of the ROC analyses for differentiating different groups. VMHC voxel-mirrored homotopic connectivity, *QUIP-RS* Questionnaire for Impulsive-Compulsive Disorders in Parkinson’s Disease Rating Scale, *PD* Parkinson’s Disease, *ICD* impulse control disorders, *ROC* receiver operating characteristic, *AUC* area under the curve, *CI* confidence interval, *HCs* healthy controls.

Correlation analysis showed a negative relationship between Questionnaire for Impulsive-Compulsive Disorders in Parkinson’s Disease rating scale (QUIP-RS) scores and mean VMHC values of the MFG in PD-ICD group (*r* = −0.625, *p* = 0.013, Fig. 2a), indicating that with the aggravation of ICD symptoms, the function coordination of the MFG became poorer.

In addition, as illustrated in Table 3 and Fig. 2b, receiver operating characteristic (ROC) analyses revealed that the area under the curve (AUC) of the MFG was 0.736 when separating PD-ICD patients from the PD-no ICD patients (95% confidence interval [CI]: 0.5954–0.8735, *p* = 0.004). Meanwhile, the AUC of the MFG was 0.802 when PD-ICD patients were separated from HCs (95% CI: 0.6885–0.9151, *p* < 0.001).

**Table 3:** ROC analyses for differentiating different groups.

Brain regions	AUC	*p* value	95% CI	Sensitivity	Specificity	Cut-off point
Middle frontal gyrus						
Separating PD-ICD from PD-no ICD	0.736	0.004^**^	0.5954–0.8735	0.667	0.788	0.1243
Separating PD-ICD from HCs	0.802	<0.001^***^	0.6885–0.9151	0.702	0.810	0.2126
Separating PD-no ICD from HCs	0.589	0.202	0.4519–0.7258	0.568	0.697	0.2468

*ROC* receiver operating characteristic, *AUC* area under the curve, *CI* confidence interval, *PD* Parkinson’s disease, *ICD* Impulse control disorders, *HCs* healthy controls.

*p < 0.05, **p < 0.01, ***p < 0.001.

## Discussion

We postulated that brain laterality may act as a key role in the pathogenesis of ICD, and hypothesized that laterality occurred primarily in brain regions associated with reward and response inhibition networks in the present study. Actually, we discovered that PD-ICD group had reduced VMHC values of MFG, relative to those without ICD symptoms. Compared to HCs, PD-ICD group principally showed decreased VMHC values in MFG, middle and superior OFG, IFG, and caudate. Further, the higher was the severity of ICD, the lower were the mean VMHC values of MFG. Besides, ROC analyses showed that the reduced VMHC values of MFG could be utilized to distinguish PD-ICD from PD-no ICD and HCs groups. However, we did not observe morphologic alterations of cortical thickness and fiber tracts among the three groups. Therefore, we supposed that interhemispheric function desynchronization was irrelevant to cortical thickness and fiber tracts alterations, and that changes in functional synchronization of ICD had not result in alteration of cortical thickness and fiber tracts. These above multiparametric magnetic resonance imaging (MRI) findings might afford a new perspective to understand the neural substrates underlying ICD.

As components of prefrontal network, the MFG, OFG, and IFG were considered as key nodes in motor inhibition network, which could suppress the reaction to incongruous stimulation by overriding the motor system’s automatic response tendency^14^. It is well known that inhibitory control was a vital executive function, involved in restraining a prepotent response or allowing proper actions to fulfill intricate task requirements and adapt to varying environment^15^. Meanwhile, the caudate was also indispensable during the successful response inhibition which was implicated in preventing the automatic execution of an action^14^. On the other hand, caudate is one of reward-related regions responding to general reward processing and its impairment could lead to be hard to inhibit aberrant behaviors in spite of the predictable negative outcomes^16,17^. Indeed, suppressing an ongoing initiated reaction could be intensely dependent on the frontal striatum pathway^18^, thus the disruption of this loop in PD-ICD patients may bring about lessening resistance to instant rewards, regardless of long-term consequences^19^. ICD in PD has previously been reported as a multidimensional concept that includes the changes of reward processing, inhibition control, learning from reward and loss, and conflict processing^20^. Although the caudate was the result of ICD compared with HCs, we did not find this brain region when comparing no-ICD with HCs. Combined with previous research findings, our results further confirmed that the pathogenesis of ICD was related to reward and inhibition systems. Moreover, it is worth noting that our results can complement previous studies, which have not emphasized the critical role of lateralization of response inhibition and reward system in ICD. In fact, the thinning of the corpus callosum was found by Roberta et al. in PD patients with ICD^21^. Concerning that the corpus callosum is the largest white matter structure connecting the two hemispheres of the brain, previous research suggested that the structure alterations of corpus callosum are associated with compromised interhemispheric functional communications^12^. Therefore, the reduced VMHC of regions primarily involved in inhibitory control and reward networks detected in our study might reflect uncoordinated inhibition control and reward processing in PD-ICD group. Hence, we hypothesized that disconnection and imbalance of impulsivity and inhibitory might be an important mechanism for inducing ICD. In accordance with our hypothesis, imaging researches of PG demonstrated right lateralized reward system alteration, specifically, the changes only detected in the right ventral striatum in answer to gambling stimuli^22^ as well as the processing of monetary reward^23^. In another study, PD-ICD patients exhibited abnormality limited to the right cerebral cortex and subcortical regions involving reward processing and inhibitory control^24^. Furthermore, we explored the correlation between VMHC values of MFG and impulsiveness symptom severity within the PD-ICD group to confirm whether connectivity-based findings were related to behavioral measures. Our findings revealed that QUIP-RS scores were negatively correlated with the mean VMHC values of MFC. That is, when patients’ clinical syndromes were more serious, their QUIP-RS scores increased, and their ICD symptoms severity might be associated with the impaired MFC function. Besides, the ROC analyses revealed that the mean VMHC values of MFG could identify the PD-ICD patients from PD-no ICD patients or HCs, which might be used as a potential feature of PD patients with ICD.

In interpreting the present findings, several limitations are worth considering. First, all of the patients underwent the MRI scanning during the “ON” medication state. Nevertheless, previous study suggested that dopaminergic treatments modulated the functional connectivity. In our investigation, the PD-ICD group had a little higher total levodopa equivalent daily dose (LEDD_TOTAL_) relative to PD-no ICD group without statistically significant. Thus, we cannot rule out the impact of pharmacological treatment on the discoveries of functional connectivity. Yet, the literature we consulted before conducting the study supported MRI scanning of ICD patients during “ON” state^24–26^. Further, ICD specific mechanism could be more likely to be detected during “ON” phase, since that ICD symptoms were closely related to dopamine replacement therapy. Technically speaking, taking medication can reduce unnecessary movements in patients and therefore limit possible movement-related artifacts. Second, the PD-ICD samples enrolled in our study were heterogeneous and could have neglected some findings specific to a single ICD category. Our finding may be driven by CS since the large proportion of it. On account of the sample size limitation, we did not perform a separate analysis for a single ICD presentation. So future studies concentrated on single ICD are urgently needed to illustrate their respective clinicoradiological correlations. Third, VMHC is methodologically limited in that it is unable to investigate the internal functional connectivity of the hemisphere or verdict which side of the brain is impaired. Fourth, the clinical data of the subjects we included did not exactly match. HAMA, HAMD-24, and MMSE scores in HCs were significantly different from those in PD with or without ICD. But when analyzing the data, we had included these as covariates to minimize their impact on the results.

In conclusion, our findings of the neural laterality of reward and inhibitory system may make further understanding of the pathophysiological mechanisms of ICD behaviors in PD.

## Methods

### Subjects

The final study included 93 participants recruiting from the Neurology Department of the First Affiliated Hospital of Nanjing Medical University, Nanjing, China. There were 23 PD patients with ICD (PD-ICD), 33 PD patients without ICD symptoms (PD-no ICD), and 37 healthy controls (HCs). Participants were matched for sex, age, intelligence, and education. All patients were diagnosed on the basis of United Kingdom Parkinson’s Disease Society Brain Bank criteria and were screened for the presence of ICD according to the Diagnostic and Statistical Manual of Mental Disorders research criteria and the Questionnaire for Impulsive-Compulsive Disorders in Parkinson’s Disease (QUIP)^27^. This diagnosis was double-confirmed during the semi-structured interview with a neurologist, using standardized criteria for PG, HS, BE, and CS^25^. In the PD-ICD group, twenty-three patients had at least one current ICD symptom that developed after PD diagnosis and initiation of dopamine replacement therapy. Further, we assessed the severity of ICD by the QUIP-RS and each ICD patients had a QUIP-RS score above the critical value for at least one ICD subtype^28,29^. Details of each PD patients’ ICD characteristics were described in Supplementary Table 2. In addition, we also confirmed that HCs and patients in PD-no ICD group had never experienced an ICD by their negative responses to QUIP. Thirty-seven controls were recruited from hospital personnel, non-consanguineous relatives and society.

Exclusion criteria were: (1) history of alcohol or drug addiction; (2) any neuropsychiatric disorders other than PD, especially severe depression, obsessive-compulsive and bipolar disorders; (3) serious cardiovascular, metabolic or neurosurgical procedures (including deep brain stimulation) or infusion therapies (duodenal levodopa or apomorphine infusion); (4) severe visual hallucinations interfering with clinical and neuropsychological assessments; (5) ICD patients were not symptomatic during examination; (6) dyskinesia complications; (7) evidence of brain anatomical abnormalities at routine MRI; (8) contraindications of MRI scans. Furthermore, all PD patients should have stable dopaminergic therapy at least two months. Prior to enrollment, approval was obtained by the ethics committee of the First Affiliated Hospital of Nanjing Medical University and written informed consent were signed by all participants.

### Demographic and clinical assessment

All PD patients were evaluated during ON phase when the dopaminergic medication was active and symptoms were well controlled. We assessed their disease stage and severity using the H-Y stage scale and the UPDRS-III, respectively. In addition, we calculated dopamine agonist equivalent daily dose (LEDD_DA_) and LEDD_TOTAL_ according to the previous literature^30^. We conducted a comprehensive assessment of the patients including mental symptoms (HAMD-24, HAMA, and BIS) and cognitive situation [Frontal Assessment Battery (FAB) and MMSE]. Demographic information including age, gender, education level, disease duration, age at diagnosis, and affected side at onset were also collected.

### MRI data acquisition

All patients underwent MRI scanning in the morning while they were still under the effect of regular dopaminergic medication dose. Meanwhile, all participants were explicitly informed to remain motionless, and not to think about anything special. MRI scans were accessed on a 3.0 T Siemens MAGNETOM Verio whole-body MRI system (Siemens Medical Solutions, Germany). Three-dimensional T1-weighted anatomical images were collected using the following volumetric 3D magnetization-prepared rapid gradient-echo (MP-RAGE) sequence: repetition time [TR] = 1900 ms, echo time [TE] = 2.95 ms, flip angle [FA] = 9°, thickness = 1 mm, slices = 160, field of view [FOV] = 230 × 230 mm^2^, acquisition matrix = 256 × 256 and voxel size = 1 × 1 × 1 mm^3^. Resting-state functional images were acquired using an echo-planar imaging (EPI) sequence with the following parameters: TR = 2000 ms, TE = 21 ms, FA = 90°, FOV = 256 × 256 mm^2^, in-plane matrix = 64 × 64, slices = 35, thickness = 3 mm, gap = 0 mm, voxel size = 3 × 3 × 3 mm^3^, total volumes = 240.

### Preprocessing of fMRI data analysis and voxel-mirrored homotopic connectivity

Rs-fMRI data preprocessing was analyzed by the Data Processing Assistant for Resting-State fMRI (DPARSF, http://www.restfmri.net/forum/dparsf) and REST (http://restfmri.net). The steps were summarized as follows: (1) discard the first ten time points and correct time differences between slice and head motion (Friston 24 parameter), (2) co-register T1 structural images to the mean EPI scans, (3) spatial normalization and re-sample to 3 × 3 × 3 mm^3^, (4) spatial smooth with a 6 mm full width half maximum Gaussian kernel, (5) filter (0.01–0.08 Hz) and remove linearly detrended, (6) regress nuisance signals [six head motion parameters, white matter, and cerebrospinal fluid (CSF) signals]. Two PD-ICD patients whose head motions exceeded 2 mm of translation or 2° of rotation throughout the scanning phase were eliminated from the experiment. Further, we calculated the mean FD of each subject and employed it as a covariate for subsequent inter-group comparisons of VMHC. The group comparisons of FD did not reveal any differences (*p* > 0.05).

REST was also applied to analyze VMHC and the detailed operation sequences have been elaborated in previous literature (9). In short, for each subject, the Pearson correlation coefficient (r) was computed between a given voxel and its counterpart voxel in the contralateral hemisphere. Then, to improve the normality of the expected statistical distribution, the correlation r values were subject to Fisher z-transformed. The resulting values constituted the VMHC and were used for comparison in the following inter-group comparisons.

Then, a voxel-based comparison of VMHC maps among the three groups was performed. For the sake of excluding the possibility that VMHC differences created by specific factors, gender, age, education level, mean FD, MMSE, HAMA, and HAMD-24 scores were input as uninterested covariates in the ANCOVA analysis. Voxels that showed significant differences in the ANCOVA analysis were then subjected to post-hoc t-tests to determine the differences between each pair (voxel level < 0.01, cluster size > 17 voxels, corresponding to a corrected *p* < 0.05 as determined by AlphaSim correction). AlphaSim is an approach which utilizes Monte Carrlo simulations to correct for multiple comparisons. Parameters included: single voxel *p* = 0.01; 1000 simulations; full width at half maximum = 6 mm; cluster connection radius *r* = 5 mm; and the mask of global gray matter.

### Cortical thickness

We further analyzed cortical thickness restricted to the brain regions showing significant differences in the VMHC analysis to explore whether these VMHC consequences were related to structural alternations. Cortical reconstruction and estimation of cortical thickness were carried out by FreeSurfer image analysis suite (http://surfer.nmr.mgh.harvard.edu/, version 6.0) on the 3D T1-weighted images^31^. The main procedures included: (1) automatic skull stripping to remove extra-cerebral structures, brainstem and cerebellum, (2) registration to Talairach space, (3) intensity normalization, (4) segmentation into white matter (WM), gray matter (GM), and CSF, (5) tessellation of the WM/GM borders, (6) automatic topology correction, (7) surface deformation to optimally set tissue boundaries^32,33^. The consequences of the segmentation process were visually censored by two clinicians who had no knowledge of participants. Then, we calculated the cortical thickness as the average shortest distance between the WM and GM surfaces at each vertex of the reconstructed cortical mantle.

To measure the degree of asymmetry in the thickness for each bilaterally cortical area, we introduced asymmetry index (AI) according to the following Eq. (1)^34^. R and L respectively represented the corresponding voxels’ cortical thickness values of the right and left hemispheres.

1 $$AI = \frac{{\left| {L - R} \right|}}{{L + R}} \times 100$$

When L was greater than R, it meant “leftward asymmetric areas”. When R was greater than L, it represented “rightward asymmetric areas”. We believed that when the AIs values of a subject with ICD or no-ICD were less than those HCs, the degree of cortical asymmetry was reduced, and vice versa. Adjusted for age, sex, degree of education, the AI values of the three groups were analyzed by ANCOVA. Here, we only analyzed the brain regions which showing significant differences in the above VMHC comparisons. Correction for multiple comparisons (Bonferroni, *p* < 0.05) was applied to threshold analyses.

### TBSS

TBSS was applied to compare fiber tracts impairment across the whole brain among three groups. The detailed content of DTI images acquisition, preprocessing and TBSS analyses was illustrated in the supplemental materials.

### Statistical analysis

The data were analyzed using IBM SPSS statistics 20.0 (Chicago, IL, USA). Sociodemographic and clinical characteristics were compared by two-sample t-test, Chi square test, Mann-Whitney test, Kruskal-Wallis test and one-way analysis of variance (ANOVA), as appropriate. The Bonferroni correction was also performed. Besides, we extracted the brain region exhibiting significant differences between the PD-ICD and PD-no ICD group as ROI. Partial correlation analysis was employed to compute correlation between the mean VMHC values of the selected ROI and the QUIP-RS scores in PD patients with ICD. LEDD, disease duration and age at onset, MMSE, HAMA, and HAMD-24 scores were included as covariates.

The extracted brain regions were further evaluated by ROC curves to assess whether they could be deemed as ICD recognition features. The specificity, sensitivity, and area AUC were described for cut-off values. Optimal cut-off was chosen by maximizing Youden’s index.

### Reporting summary

Further information on research design is available in the Nature Research Reporting Summary linked to this article.

## Supplementary information

Supplementary Information

Reporting Summary

## Data Availability

The data that support the findings of this study are available from the corresponding author upon reasonable request.
