# Supplementary material for: Abnormal interhemispheric resting state functional connectivity in Parkinson’s disease patients with impulse control disorders
Source: NPJ Parkinsons Dis. 2021 Jul 16;7:60. doi: 10.1038/s41531-021-00205-7 (PMC8285494; doi:10.1038/s41531-021-00205-7)
Supplement: Supplementary file 1 — Supplementary Information [file 41531_2021_205_MOESM1_ESM.pdf]

Supplemental Material

Image acquisition

DTI images were acquired using spin echo planar imaging sequence. Parameters were as follows: TR = 9800 ms, TE = 95 ms, FOV = 256 × 256 mm^2, number of excitations (NEX) = 1, matrix = 128 × 128, slice thickness = 2 mm and slice gap = 0 mm. Diffusion gradients were applied in 30 non-collinear directions with a b factor of 1000 s/mm^2 after an acquisition without diffusion weighting (b = 0 s/mm^2) for reference.

Image processing and statistical analysis

The preprocessing of diffusion images roughly included: converting from DICOM to NIFTI, realignment, eddy current and motion artifact correction, obtaining fractional anisotropy (FA) and mean diffusivity (MD) maps.

TBSS pipeline was used to compare DTI metrics among the three groups. The procedures included the following steps: (1) FA images were nonlinearly aligned to FMRIB-58 FA map from Montreal Neuroimaging Institute (MNI) template; (2) the mean FA image was created and the mean FA skeleton was generated, with the threshold at 0.2; (3) individual FA and MD data were projected onto the FA skeleton. The voxel-wise statistics were performed through a general linear model for skeletonized FA and MD images. This analysis used the randomized permutation test (5000 permutations) for multiple comparisons correction and a threshold-free cluster enhancement (TFCE). Sex, age, education level, Mini Mental State Examination (MMSE), Hamilton Anxiety Scale (HAMA), and Hamilton Depression Scale-24 (HAMD-24) scores were used as nuisance covariates. Results were considered significant at p value < 0.05, TFCE-corrected for multiple comparisons. These above steps were performed using the PANDA toolbox (https://www.nitrc.org/projects/panda)

based on FMRIB Software Library (FSL 5.0; https://www.nitrc.org/projects/fsl). However, no significant differences were detected among the groups, as shown in the following Supplementary Figure 1.

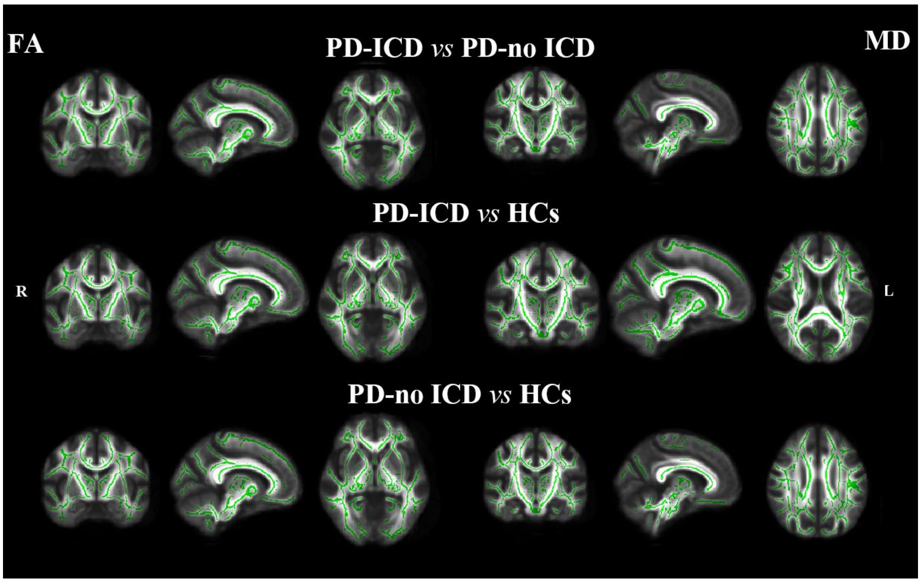

Supplementary Figure 1. TBSS group comparison results.

The TBSS skeleton is depicted in green. The threshold for display was set to $p < 0.05$ (corrected). Abbreviations: PD: Parkinson's Disease; TBSS: tract-based spatial statistics; FA: fractional anisotropy; MD: Mean diffusivity; Parkinson's Disease; ICD: Impulse control disorders; HCs: health controls; R: right; L: left.

Supplementary Table 1. Asymmetry index mean values of the brain regions that showing significant differences of VMHC among three groups.

Brain regions	Asymmetry Index			
	PD-ICD	PD-no ICD	HCs	p values
Middle frontal gyrus	1.48 ± 0.45	1.24 ± 0.19	1.10 ± 0.14	0.961
Middle orbital frontal gyrus	2.50 ± 0.68	2.10 ± 0.34	2.01 ± 0.22	0.799
Inferior frontal gyrus(pars opercularis)	1.75 ± 0.39	2.11 ± 0.30	1.83 ± 0.25	0.726
Middle temporal gyrus	1.37 ± 0.16	1.60 ± 0.26	1.37 ± 0.17	0.839
Superior temporal gyrus	2.54 ± 2.08	1.46 ± 1.09	1.72 ± 1.12	0.086
Precental gyrus	2.06 ± 0.53	2.09 ± 0.47	2.85 ± 0.57	0.492
Angular gyrus	1.47 ± 0.20	1.75 ± 0.25	1.83 ± 0.20	0.574
Superior orbital frontal gyrus	0.98 ± 0.24	1.03 ± 0.13	1.08 ± 0.14	0.671
Postcentral gyrus	1.40 ± 0.24	1.70 ± 0.23	2.62 ± 0.39	0.138

Values are represented as the mean ± standard deviation. The significance of ANCOVA was set at $p < 0.05$. Abbreviation: ICD: Impulse control disorders; HCs: healthy controls.

Supplementary Table 2. Detailed information on patients with ICD.

Subjects	Sex (M/F)	Age (y)	Main ICD	Other ICD-RD	DA
PD-ICD 1	M	63	CS	-	Pramipexole
PD-ICD 2	F	63	PG	-	-
PD-ICD 3	M	67	CS	-	Pramipexole
PD-ICD 4	F	62	CS	-	Piribedil
PD-ICD 5	M	66	CS	-	Pramipexole
PD-ICD 6	M	73	BE	CS, Hobbyism-punding	Piribedil
PD-ICD 7	F	60	CS	-	Pramipexole
PD-ICD 8	M	48	PG	-	Pramipexole
PD-ICD 9	F	74	PG	-	Piribedil
PD-ICD 10	F	54	PG	-	Pramipexole
PD-ICD 11	F	64	BE	-	Pramipexole
PD-ICD 12	M	59	CS	-	Piribedil
PD-ICD 13	M	62	BE	-	Pramipexole
PD-ICD 14	F	50	CS	-	Pramipexole
PD-ICD 15	F	32	PG	-	-
PD-ICD 16	M	61	BE	-	Piribedil
PD-ICD 17	M	52	BE	-	Pramipexole
PD-ICD 18	M	52	HS	-	-
PD-ICD 19	M	48	HS	-	Piribedil
PD-ICD 20	F	65	BE	-	Pramipexole
PD-ICD 21	M	64	CS	DDS	Pramipexole

Abbreviation: M = male; F = female; y: year; ICD: impulse control disorders; ICD-RD: impulse control and related disorders; BE = binge eating; HS = hypersexuality; CS = compulsive shopping; PG = pathological gambling; DDS: dopamine dysregulation syndrome; DA = dopamine agonist. All patients were also taking levodopa.
