# Supplementary material for: Abnormal interhemispheric resting state functional connectivity in Parkinson’s disease patients with impulse control disorders
Source: NPJ Parkinsons Dis. 2021 Jul 16;7:60. doi: 10.1038/s41531-021-00205-7 (PMC8285494; doi:10.1038/s41531-021-00205-7)
Supplement: Supplementary file 2 — Reporting Summary [file 41531_2021_205_MOESM2_ESM.pdf]

nature research

Corresponding author(s): NPJPARKD-00649R1

Last updated by author(s): Jun 11, 2021

Reporting Summary

Nature Research wishes to improve the reproducibility of the work that we publish. This form provides structure for consistency and transparency in reporting. For further information on Nature Research policies, see our Editorial Policies and the Editorial Policy Checklist.

Statistics

For all statistical analyses, confirm that the following items are present in the figure legend, table legend, main text, or Methods section.

n/a	Confirmed	
☐	☒	The exact sample size (*n*) for each experimental group/condition, given as a discrete number and unit of measurement
☐	☒	A statement on whether measurements were taken from distinct samples or whether the same sample was measured repeatedly
☐	☒	The statistical test(s) used AND whether they are one- or two-sided<br>*Only common tests should be described solely by name; describe more complex techniques in the Methods section.*
☐	☒	A description of all covariates tested
☐	☒	A description of any assumptions or corrections, such as tests of normality and adjustment for multiple comparisons
☐	☒	A full description of the statistical parameters including central tendency (e.g. means) or other basic estimates (e.g. regression coefficient) AND variation (e.g. standard deviation) or associated estimates of uncertainty (e.g. confidence intervals)
☐	☒	For null hypothesis testing, the test statistic (e.g. *F*, *t*, *r*) with confidence intervals, effect sizes, degrees of freedom and *P* value noted<br>*Give P values as exact values whenever suitable.*
☒	☐	For Bayesian analysis, information on the choice of priors and Markov chain Monte Carlo settings
☐	☒	For hierarchical and complex designs, identification of the appropriate level for tests and full reporting of outcomes
☐	☒	Estimates of effect sizes (e.g. Cohen's *d*, Pearson's *r*), indicating how they were calculated

Our web collection on statistics for biologists contains articles on many of the points above.

Software and code

Policy information about availability of computer code

Data collection	no custom algorithms or software were used
Data analysis	no custom algorithms or software were used

For manuscripts utilizing custom algorithms or software that are central to the research but not yet described in published literature, software must be made available to editors and reviewers. We strongly encourage code deposition in a community repository (e.g. GitHub). See the Nature Research guidelines for submitting code & software for further information.

Data

Policy information about availability of data

All manuscripts must include a data availability statement. This statement should provide the following information, where applicable:

- Accession codes, unique identifiers, or web links for publicly available datasets
- A list of figures that have associated raw data
- A description of any restrictions on data availability

The data that support the findings of this study are available from the corresponding author upon reasonable request.

Field-specific reporting

Please select the one below that is the best fit for your research. If you are not sure, read the appropriate sections before making your selection.

☒ Life sciences ☐ Behavioural & social sciences ☐ Ecological, evolutionary & environmental sciences

For a reference copy of the document with all sections, see nature.com/documents/nr-reporting-summary-flat.pdf

Life sciences study design

All studies must disclose on these points even when the disclosure is negative.

Sample size	We enrolled 21 PD patients with ICD, 33 PD patients without ICD and 37 health controls. The incidence of Parkinson's disease is about 1.7%, and ICD occurred in 6.1%-31.2% PD patients. We think our sample size are sufficient.
Data exclusions	Two participants (2 PD patients with ICD) with head motions more than 2.0 mm of translation or 2.0° of rotation were excluded.
Replication	All attempts at replication were successful.
Randomization	We collected all participants information, including age, gender, education, and disease duration. And further used them as covariates during the analyses.
Blinding	Our study aimed to study the neural mechanisms of ICD in Parkinson's disease. And the investigators were grouped according to their own disease status.

Reporting for specific materials, systems and methods

We require information from authors about some types of materials, experimental systems and methods used in many studies. Here, indicate whether each material, system or method listed is relevant to your study. If you are not sure if a list item applies to your research, read the appropriate section before selecting a response.

Materials & experimental systems

n/a	Involved in the study	
☒	☐	Antibodies
☒	☐	Eukaryotic cell lines
☒	☐	Palaeontology and archaeology
☒	☐	Animals and other organisms
☐	☒	Human research participants
☒	☐	Clinical data
☒	☐	Dual use research of concern

Methods

n/a	Involved in the study	
☒	☐	ChIP-seq
☒	☐	Flow cytometry
☐	☒	MRI-based neuroimaging

Human research participants

Policy information about studies involving human research participants

Population characteristics	For PD patients with ICD, 9 were females and 12 were males. Their average was 59 and all of them were treated with dopamine drugs. For PD patients without ICD, 13 were females and 20 were males. Their average was 61.7 and all of them were treated with dopamine drugs. For health controls, 12 were females and 25 were males. Their average was 62.0 and none of them were treated with dopamine drugs.
Recruitment	Thirty-seven controls were recruited from hospital personnel, non-consanguineous relatives and society. Patients were recruited from outpatient patients who went to the hospital for treatment.
Ethics oversight	This study was approved by the ethics committee of the First Affiliated Hospital of Nanjing Medical University.

Note that full information on the approval of the study protocol must also be provided in the manuscript.

Magnetic resonance imaging

Experimental design

Design type	resting-state

Design specifications	All patients underwent MRI scanning in the morning while they were still under the effect of regular dopaminergic medication dose. For health controls, they also underwent MRI scan.
Behavioral performance measures	All PD patients were evaluated during ON phase when the dopaminergic medication was active and symptoms were well controlled. We assessed their disease stage and severity using the H-Y stage scale and the UPDRS-III, respectively. In addition, we conducted a comprehensive assessment of the patients including mental symptoms (HAMD-24, HAMA and BIS) and cognitive situation [Frontal Assessment Battery (FAB) and MMSE].

Acquisition

Imaging type(s)	functional,structural
Field strength	3.0 T
Sequence & imaging parameters	Three-dimensional T1-weighted anatomical images were acquired using the following volumetric 3D magnetization-prepared rapid gradient-echo (MP-RAGE) sequence with the following parameters: repetition time [TR] = 1900 ms, echo time [TE] = 2.95 ms, flip angle [FA] =9°, slice thickness = 1 mm, slices = 160, field of view [FOV] = 230 × 230 mm2, matrix size = 256 × 256 and voxel size = 1 × 1 × 1 mm3. Resting-state functional images were collected using an echo-planar imaging (EPI) sequence with the following parameters: TR = 2000 ms, TE = 21 ms, FA = 90°, FOV = 256 × 256 mm2, in-plane matrix = 64 × 64, slices = 35, slice thickness = 3 mm, no slice gap, voxel size = 3 × 3 × 3 mm3, total volumes = 240.
Area of acquisition	whole brain scan

Diffusion MRI ☒ Used ☐ Not used

Parameters	DTI images were acquired using spin echo planar imaging sequence. Parameters were as follows: TR = 9800 ms, TE = 95 ms, FOV = 256 × 256 mm2, number of excitations (NEX) = 1, matrix = 128 × 128, slice thickness = 2 mm and slice gap = 0 mm. Diffusion gradients were applied in 30 non-collinear directions with a b factor of 1000 s/mm2 after an acquisition without diffusion weighting (b = 0 s/mm2) for reference.

Preprocessing

Preprocessing software	MATLAB software (version R2016b, Math Works, Inc., Natick, MA, USA)<br>DPARSF, http://www.restfmri.net/forum/dparsf<br>REST, http://restfmri.net<br>FreeSurfer
Normalization	DARTEL normalization was applied to compute the transformations from the native space to the Montreal Neurological Institute (MNI) space.
Normalization template	MNI space
Noise and artifact removal	Several sources of spurious variance were regressed out, including the white matter signal, the cerebral spinal fluid signal, and six head motion parameters obtained by head-motion correction.
Volume censoring	SPM12 toolbox (http://www.fil.ion.ucl.ac.uk/spm/)

Statistical modeling & inference

Model type and settings	Case controlled study
Effect(s) tested	have used ANOVA analysis

Specify type of analysis: ☒ Whole brain ☐ ROI-based ☐ Both

Statistic type for inference<br>(See Eklund et al. 2016)	Voxel level ⍰ 0.01, cluster size ⍰ 17 voxels, corresponding to a corrected p ⍰ 0.05 as determined by AlphaSim correction. AlphaSim parameters included: single voxel p = 0.01; 1000 simulations; full width at half maximum = 6 mm; cluster connection radius r = 5 mm; and the mask of global gray matter.
Correction	Monte Carlo

Models & analysis

n/a	Involved in the study
☐	☒ Functional and/or effective connectivity
☒	☐ Graph analysis
☒	☐ Multivariate modeling or predictive analysis

Functional and/or effective connectivity	Pearson correlation
